# CO_2_ Laser-Assisted Deep Sclerectomy Surgery Compared with Trabeculectomy in Primary Open-Angle Glaucoma: Two-Year Results

**DOI:** 10.1155/2021/6639583

**Published:** 2021-02-10

**Authors:** Hengli Zhang, Yizhen Tang, Xiaowei Yan, Lihua Ma, Yulei Geng, Fan Li, Guangxian Tang

**Affiliations:** ^1^Department of Ophthalmology, Shijiazhuang People's Hospital, Shijiazhuang, Hebei 050000, China; ^2^Department of Ophthalmology and Visual Science, Eye Institute, Eye & ENT Hospital, Shanghai Medical College, Fudan University, Shanghai 200031, China

## Abstract

**Purpose:**

To compare the effectiveness and safety of carbon dioxide (CO_2_) laser-assisted deep sclerectomy surgery (CLASS) and trabeculectomy (Trab) for treatment of primary open-angle glaucoma (POAG).

**Methods:**

In this retrospective and comparative study, 77 eyes of 62 patients with POAG were studied and divided into the CLASS and Trab groups. The best-corrected visual acuity (BCVA), intraocular pressure (IOP), number of medications, surgical success rate, and complications were analyzed.

**Results:**

The mean follow-up periods were 27.89 ± 2.94 months and 26.11 ± 2.06 months in the CLASS and Trab groups, respectively. 30 eyes (24 patients) underwent CLASS and 47 eyes (38 patients) underwent Trab. The BCVA in the CLASS and Trab groups was recovered to baseline at postoperative 1 week and 1 month, respectively. At last follow-up visits, a remarkable reduction in the IOP and number of medications was observed in both groups, and no significant difference was found in those between the two groups. The complete success rates were 51.7% and 47.7% in postoperative 24 months in the CLASS and Trab groups, respectively (*P* > 0.05). There were higher rates of delayed anterior chamber formation (21.3%) and thin-wall filtrating blebs (10.6%) in the Trab group. Meanwhile, the peripheral anterior synechiae were only observed in the CLASS group, and the ratio was 30%.

**Conclusions:**

CLASS is an effective and safe treatment modality for POAG, with fewer filtering bleb-related complications and quicker visual recovery in the early postoperative stage than trabeculectomy. The efficacy of lowering intraocular pressure was similar for both procedures.

## 1. Introduction

Trabeculectomy (Trab) is considered a classical surgical approach and the gold standard surgery for treating primary open-angle glaucoma (POAG) [1]. Nevertheless, it is associated with early postoperative complications: hypotonia-related complications such as shallow anterior chamber and detachment of the choroid and filtering bleb-related complications such as thin wall, leakage, scarring, infection, and endophthalmitis [2–4]. Furthermore, filtering bleb-independent procedures such as the nonpenetrating trabecular surgery (NPTS) have not been widely used owing to the long learning curve and technical challenges, although it is considered an attractive approach considering its safety profile. The carbon dioxide (CO_2_) laser-assisted sclerectomy surgery (CLASS) is the optional surgical approach for treating patients with glaucoma. This surgery is mainly suitable to treat those with open-angle glaucoma [5, 6]. This technique does not require anterior chamber penetration and intraocular tissue dissection, thereby reducing early complications and filtering bleb-related complications that are common after trabeculectomy surgery. CLASS is simpler and has a shorter learning curve and surgical time than NPTS. As the laser energy is controllable, the depth and size of the scleral pool can be well controlled by adjusting the laser energy and lasing scope, leading to more accurate processing of the Schlemm canal and trabeculo-Descemet membrane window (TDW). In addition, the laser can be absorbed and its effect can be terminated when the aqueous humor percolates. Therefore, the probability of TD membrane (TDM) perforation is extremely low during the operation, thereby improving the safety and surgical success rate [7]. According to the studies by Geffen and Greifn et al., CLASS is comparable to NPDS in terms of lowering the intraocular pressure (IOP) and the number of medications during the one-year and two-year follow-up periods [5, 7]. However, except for Jankowska-Szmul et al.'s study [8], few studies have compared the effects and safety of CLASS vs. trabeculectomy for open-angle glaucoma. Therefore, this study has aimed to compare the efficacy and safety of CLASS and Trab for the treatment of POAG.

## 2. Materials and Methods

### 2.1. Patients

The inclusion criteria were patients aged ≥18 years with the following: POAG [9], medically uncontrolled IOP, and no previous intraocular surgery. The exclusion criteria included patients with other ocular disorders such as severe meibomian gland dysfunction, secondary ocular inflammation, or opacity that might interfere with optic nerve evaluation. In this retrospective and comparative study, 77 eyes from 62 patients with POAG hospitalized in the Shijiazhuang People's Hospital were enrolled between January 2017 and January 2018. Thirty eyes from 24 patients underwent CLASS (CLASS group) and 47 eyes from 38 age- and sex-matched patients underwent trabeculectomy (Trab group). The information of all patients before surgery is shown in Table 1. No significant difference from the baseline data was found between the two groups (*P* > 0.05). All diagnoses of POAG followed the guidelines of the International Society of Geographical and Epidemiological Ophthalmology. In addition, all surgeries were performed by two experienced ophthalmologists.

### 2.2. Surgical Procedure

All procedures were performed under topical anesthesia with proparacaine hydrochloride eye drops and subconjunctival anesthesia with 0.2 mL of 2% lidocaine.

#### 2.2.1. CLASS

The fornix-based conjunctival flap was used. A scleral flap measuring 5 × 5 mm^2^ with one-third scleral thickness was dissected 1.5 mm anterior to the corneal limbus for thorough exposure of the TDM. Under the conjunctiva and scleral flap, a piece of sponge soaked in 0.2–0.4 mg/mL mitomycin was applied for 3-4 min and then rinsed with 100 mL of balanced salt solution. Subsequently, the CO_2_ laser system (OT-134-IOPtiMate; IOPtima Ltd., Ramat Gan, Israel) with the desired scanning dimensions and shape (marked with red laser beam) that could be altered in the range of 4 × 3 mm was used to repeatedly ablate >90% scleral tissue until the suprachoroidal space was detected and the residual scarring issue was removed. Mitomycin was applied again to the scleral pool for 1 min, which was washed with 100 mL of balanced salt solution. Following that, CO_2_ laser was applied to ablate the TDM area, outer wall of Schlemm's canal, and trabecular meshwork until a slow and continuous percolation of aqueous humor was seen; the ablated area measured about 4 × 1 mm in width. The laser energy was set to and maintained at 21–18 W for all cases, and the interval between the laser applications was 2-3 s to detect continuous aqueous humor percolation of the ablated area. Then, the scleral flap was sutured with 10-0 nylon sutures. Finally, the conjunctiva was fixed with 10-0 nylon sutures.

#### 2.2.2. Trabeculectomy

Trabeculectomy was conducted in the superior quadrant according to the surgical procedure described in a previous study [10]. A scleral flap of 4 × 4 mm with about one-half scleral thickness was created, and a sponge soaked in 0.2–0.4 mg/mL mitomycin was applied under the flap followed by washing with 100 mL Ringer's solution. Subsequently, paracentesis, sclerostomy, and peripheral iridectomy were performed, and the scleral flap was sutured using two stitches with a 10-0 nylon thread. The filtering function was detected, and the anterior chamber was rebuilt. Finally, the conjunctiva was sutured.

### 2.3. Observational Indicators

The following parameters were examined and analyzed before surgery, at 1 week, and at 1, 3, 6, 12, and 24 months after the surgery. The best-corrected visual acuity (BCVA) was measured with the Snellen chart, and the results were described with the logarithmic minimum angle of resolution (logMAR). IOP was measured with a calibrated Goldman applanation tonometer. Gonioscopy (a single mirror Gonio diagnostic lens), number of medications, surgical success rate, and complications were recorded. All examinations were performed by experienced ophthalmologists and technicians.

In the CLASS group, the patients underwent preoperative routine ultrasound biomicroscopy (UBM) [11] (300, Meda Co., Ltd., Tianjin, China) examination, except for gonioscopy, to assess the width and opening and closing of the full angle, especially the 12 o'clock angle. For patients with narrow angles (Shaffer's grade ≤2 in 3 or more quadrants, and the width of anterior chamber angle was 20°) [12–14], the gonioscopy showed that all of their ciliary body bands were visible under the dynamic condition, a 532-nanometer-laser peripheral iridoplasty (LPI) at 12 o'clock iris was performed, and therapeutic intervention with yttrium-aluminum-garnet (YAG) laser peripheral iris boring, which should be as close as possible to the peripheral iris and surgical site, was performed before the surgery (usually > 48 h prior to operation) to prevent postoperative peripheral anterior synechiae (PAS). The laser energy of LPI was initially 150–200 mW, the exposure time was 0.5 s, and the size of the spot was 500 *μ*m. The laser energy was adjusted according to the iris reaction that the iris was atrophied, but no bubbles were formed. The energy for laser peripheral iris boring was 4–8 mJ, the exposure time was 11 ns, and the laser hole was >200 *μ*m. Furthermore, seven eyes (23.33%) preoperatively underwent LPI combined with the YAG laser boring hole as close as possible to the peripheral iris, just corresponding to the surgical site. All manipulations were done by experienced ocular technicians.

### 2.4. Surgical Success Rate

Complete success referred to a final IOP between 6 and 18 mm Hg, without the application of antiglaucoma drugs. Qualified success referred to IOP between 6 and 18 mm Hg after the local application of antiglaucoma drugs. Surgical failure referred to two consecutive measurements of IOP of more than 18 mm Hg after topical application of ≥3 antiglaucoma drugs [8, 15].

### 2.5. Postoperative Management

All patients were postoperatively prescribed tobramycin dexamethasone drops four times a day and tobramycin dexamethasone ointment once a day for 4 weeks. The medication frequency was gradually decreased due to the relief of ocular inflammatory reaction during the follow-up period. In the CLASS group, pilocarpine was prescribed postoperatively four times a day for 3 months to prevent PAS. If PAS was formed, iridoplasty was performed using the 532 nm laser at an energy of 150–200 mW and exposure time 0.5 s to cauterize the adhesive region. Subsequently, the YAG laser goniopuncture (GPT) was performed if PAS still existed (YAG laser with the initial setting of 2–4 mJ was used to treat PAS and create a microhole in TDW for augmenting aqueous flow) [16, 17]. For patients with iris incarceration in TDW and no successful repositioning with all the aforementioned methods, needling was performed, wherein a 23 G needle was inserted into the anterior chamber at the clear corneal limbus to treat PAS and reposition the incarcerated iris. GPT and needling were not regarded as failures or complications because both are often used as normal postoperative therapies, which is essential to retain or enlarge the operative effects of glaucoma surgeries [15, 17].

### 2.6. Statistical Analysis

SPSS 19.0 (IBM, IL, USA) was used for all statistical analyses. Patients' information of both groups before surgery was compared and analyzed using unpaired *t-*test, except for the gender composition ratio that was compared using chi-square test (*χ*^2^). Repeated-measures analysis of variance (ANOVA) was used to analyze the changes in BCVA, IOP, and number of medications from preoperative baseline to 24 months between the two groups. Success rates between the two groups were compared with chi-square test (*χ*^2^). The postoperative complications were compared using Fisher's exact test. The cumulative probability of success was illustrated using Kaplan–Meier survival curves, and the Log-rank test was performed for group comparisons. A *P* value less than 0.05 was considered statistically significant.

## 3. Results

After the surgery, patients were followed up for 27.89 ± 2.94 months in the CLASS group and 26.11 ± 2.06 months in the Trab group. One patient in the CLASS group experienced severe hematencephalon after 3 months and was lost to follow-up. In the Trab group, three patients were lost to follow-up after 6 months, 1 year, and 2 years, respectively.

### 3.1. BCVA

As shown in Table 1, there was no statistical difference in the BCVA before operation (*P*=0.67). In 1 week and 1 month after surgery, the BCVA in the CLASS group was 0.28 ± 0.22 and 0.27 ± 0.24, while in the Trab group it was 0.27 ± 0.24 and 0.31 ± 0.31. Repeated-measures analysis showed the Mauchly sphericity test *P* < 0.01, and the Greenhouse–Geisser estimation was conducted (time effect, *F* = 23.14, *P* < 0.001; group effect, *F* = 1.39, *P*=0.24; time *∗* group, *F* = 12.33, *P* < 0.001, so a simple effect analysis of the data was further performed). Interestingly, the BCVA in CLASS group was significantly improved compared to Trab group at 1 week after the operation (*P*=0.02); no statistical difference was found 1 month after the operation (*P*=0.55). These data suggested that the BCVA was restored to the preoperative level in the CLASS group one week after surgery. In the Trab group, however, the BCVA was reduced 1 week after operation and gradually recovered to the preoperative level 1 month after surgery (Table 2).

### 3.2. IOP

Mean IOP remarkably decreased from (36.43 ± 6.62 mm Hg) in the CLASS group and (37.87 ± 5.74 mm Hg) in the Trab group preoperatively to (15.36 ± 3.74 mm Hg) and (16.34 ± 3.25 mm Hg) postoperatively at 24 months, respectively (repeated-measures ANOVA, *F* = 360.11, *P* < 0.001). No significant difference of group effect was observed (repeated-measures ANOVA, *F* = 0.28, *P*=0.42; Table 3). This suggested no significant difference in IOP between the two groups.

### 3.3. Medications

Mean number of medications significantly decreased from (3.57 ± 1.19) in the CLASS group and (3.60 ± 0.99) in the Trab group preoperatively to (1.31 ± 1.54) and (1.30 ± 1.32) postoperatively at 24 months, respectively (repeated-measures analysis, *F* = 108.11, *P* < 0.001). However, no significant difference in the follow-up doses of medications was found between the two groups (repeated-measures analysis, *F* = 0.05, *P*=0.83; Table 3).

### 3.4. Therapeutic Outcomes

**Figure 1:**
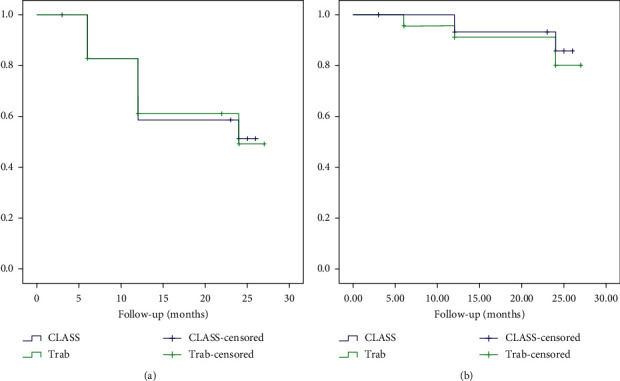
(a) Kaplan–Meier cumulative survival curves of complete success rate for the CLASS group and Trab group. (b) Kaplan–Meier cumulative survival curves of qualified success rate for the CLASS and Trab groups.

As shown in Table 4, no significant difference was found in the complete and qualitied success rates between two groups. Figures 1(a) and 1(b) show the Kaplan–Meier survival plots; the cumulative probabilities of complete and quailed success rates were not significantly different in the CLASS and Trab groups, respectively (*P*=0.94, 0.68; Log-rank test).

During the following visits, four eyes in the CLASS group with uncontrolled IOP finally underwent gonioscopy-assisted transluminal trabeculectomy, and six eyes in the Trab group underwent reoperation, including two eyes that underwent trabeculectomy and four eyes that underwent trabeculectomy combined with cataract surgery.

### 3.5. Complications and Postoperative Interventions

#### 3.5.1. CLASS Group

As listed in Table 5, one eye experienced superficial detachment of the ciliary body, which automatically recovered after 2 weeks. Nine eyes (30%) had PAS within 3 weeks after the surgery. Among them, LPI was performed on one eye with small-scope PAS (3.33%) five days after surgery, and the IOP decreased from 21 mmHg to 13 mmHg. GPT was performed on six eyes (20%) with PAS within postoperative 3 weeks, and the IOP reduced from 27.75 ± 6.92 mmHg to 11.88 ± 2.30 mmHg. Meanwhile, LPI combined with GPT was found in two eyes that showed iris incarcerated in the TDW (IOP range: 35–41 mmHg, from 11 to 9 mmHg before and after the operation) within the postoperative 3 weeks. In addition, six eyes (21.7%) with a mean IOP of 33.17 ± 6.31 mmHg encountered needling with MMC injection one year after surgery; at last, 4 eyes among them accepted the surrey again. During the follow-up period, no other complication was observed. However, two eyes underwent iris incarceration within postoperative 3 weeks, of which one eye was treated with needling while the other eye with iris microincarceration required LPI combined with GPT. Hence, the IOP was stably maintained at about 12 mmHg.

#### 3.5.2. Trab Group

GPT and LPI were not performed in the Trab group. The formation of the anterior chamber was delayed in 10 (21.3%) eyes in the early stage after the surgery. Two eyes recovered after anterior chamber reformation, and the rest recovered after receiving medical conservative therapy during the follow-up. Anterior chamber hemorrhage occurred in four eyes, and the hemorrhage was absorbed 2 weeks after the surgery with no further intervention. Choroidal detachment took place in five eyes, of which four recovered after one-week conservative therapy and the other eye recovered after draining fluid from the suprachoroidal space. One eye suffered low-tension maculopathy and gradually recovered after injecting autologous blood into the filtration bleb. The intervention of bleb needling with MMC injection was performed on 13 eyes (28.9%), one year after surgery. During the follow-up visits, a functional thin-wall filtration bleb was found in five eyes with well-controlled IOP.

## 4. Discussion

The far-infrared radiation of the CO_2_ laser (wavelength: 10600 nm) is highly effective in ablating the deep scleral tissue and outer wall of Schlemm's canal. Under a scleral flap, the laser not only ablates the TDM but also keeps the inner wall of the Schlemm canal and trabecular meshwork intact. Subsequently, the scleral pool, intrascleral (suprachoroidal) space, and TDW are constituted, which help to reduce the aqueous humor outflow resistance and ensure continuous drainage of the aqueous humor from the anterior chamber to the scleral pool and suprachoroidal space, thereby decreasing the intraocular pressure (IOP). In addition, the coagulation ablation effect of CO_2_ laser not only plays a role in hemostasis and blood vessel blockade but also reduces the risk of scleral reservoir scarring after surgery [15].

As shown in this study, the postoperative IOP significantly decreased as compared with the preoperative level in both groups (*P* < 0.001), while the variation in IOP tended to stabilize with prolonged observation time, with no significant intergroup difference in IOP 24 months after the surgery (*P* > 0.05). The number of antiglaucoma medications markedly decreased in both groups up to 24 postoperative months (*P* < 0.001). Compared with the Trab group, there were no significant differences in the follow-up doses of medication in the CLASS group. This result suggests the same efficacy for the CLASS and Trab groups on lowering the IOP. Skaat et al. studied 15 eyes with open-angle glaucoma treated using CLASS. The results at one year after the surgery showed that CLASS could safely and effectively decrease IOP in patients with POAG and exfoliative glaucoma, with a complete success rate of 45.5% following surgery for 12 months (76.9% patients used MMC intraoperatively) [17]. In a Chinese study, Yick et al. achieved a success rate of about 81.8% by investigating the one-year outcome in 23 eyes with advanced glaucoma after CLASS [18]. Geffen et al. showed that the complete success rates following CLASS after 12 and 24 months were 60.2% and 57.9%, respectively (88.8% procedures used MMC intraoperatively), and consistent with the results of the current study [5]. Another study revealed that CLASS had similar curative effects as traditional trabeculectomy [19, 20]. These investigations further validate the aforementioned beliefs and conclusions. However, the study by Jankowska-Szmul et al. wherein surgery was performed without MMC showed varied results, in that one year after surgery, the complete success rates were 35% for CLASS and 60% for Trab. The mean reduction rate in the CLASS group was lower than that in the Trab group [8]. This is inconsistent with the above reports and may be related to the absence of MMC during the operation. Other studies have reported that intraoperative use of MMC can improve the success rate and result in lower IOP level [2, 21, 22].

The present study indicated that BCVA in the CLASS group was restored to the preoperative level one week after surgery. However, in the Trab group, the BCVA was reduced and gradually restored to the baseline level, one month after surgery. The reasons for this might be as follows: (1) The anterior chamber was not penetrated during CLASS surgery, leading to the formation of a barrier by the intraocular tissue and a small fluctuation in the intraoperative and postoperative anterior chamber and IOP, which maintained the depth and stability of the anterior chamber [7]. (2) Adjustable suture was not implemented during the CLASS procedure, thus reducing the suture-induced refractive changes. (3) As intraocular tissue resection was not required, less inflammatory response was seen, consequently speeding up the recovery process.

We found that the early complications in the Trab group mainly included shallow anterior chamber (21.3%), choroid detachment (10%), and hyphema (8.5%); relative to the CLASS group, no complication was observed. Other investigations have presented a similar result (42%) for complications following trabeculectomy [23]. As expected from the opinion for CLASS, that consists in keeping a natural tissue barrier that maintains the depth of anterior chamber and impedes the occurrence of these complications [2]. The CLASS group showed a higher incidence of the adverse effect PAS than the Trab group, with nine eyes (30%) having increased IOP owing to different degrees of PAS about 3 weeks after the surgery. The relatively high rates of PAS after CLASS were also found in the following researches: Geffen et al. showed 5.6%, Cutolo et al. showed 14%, and Greifner et al. reported 26%; however, all these results are lower than that in the current research [2, 7]. The PAS might be related to the following factors: (1) The TDM is narrower and thinner than the traditional NTPS, with a position focused on the Schlemm canal's area and the pigmented trabecular meshwork under it; TDM is also nearer to the iris root, which increases the risk of PAS [7]. (2) PAS could be bound with the intraoperative dimensions and laser energy. In addition, a standardization of the ablation in CLASS procedure has been presented [7]. Thus, all operations should strictly abide by the standards, and we strongly advise the use of lower laser energy during the operation, especially for TDM ablation (laser energy: 18 W, interval: 3 s), because we speculate that the high energy might lead to thermal injury to the issues connected to the ablated area and may represent a stimulus for inflammation. If this inflammatory response was not well controlled, it may be involved in the formation of PAS. Hence, it is very important to strengthen topical anti-inflammatory treatment during the perioperative period. (3) The intraoperative and postoperative overfiltration increased the outflow and promoted PAS. Accordingly, the suture of the repositioned scleral flap was needed to suture *in situ*, ensure moderate tightness, and prevent overfiltering, which is very important to impede the PAS. (4) Seider et al. and He et al. [13, 24] suggested a high prevalence of narrow angles in the mainland population (36.9% among those aged >50 years). This may be another reason for the high incidence of PAS in this study. Patients with primary narrow-open-angle glaucoma are prone to PAS and hence should be given appropriate intervention therapy preoperatively. In our study, seven eyes with narrow angle were managed with YAG laser and 532 laser-formed laser bore in closer proximity to the peripheral iris root positioning the upper surgical area before surgery. Postoperatively, six of seven eyes did not show PAS; the remaining one eye had PAS owing to the surgical position and the laser bore not being close enough to the peripheral iris root. To reduce the incidence of postoperative PAS in patients with CLASS, pilocarpine eye drops should be routinely applied to contract the pupil; if signs of PAS are found, the dose should be increased to pull back the iris and avoid PAS [7]. In our study, in the two eyes undergoing iris incarceration within postoperative 3 weeks, one eye was treated with needling and the other eye with microincarceration was treated with LPI combined with GPT. Thus, the IOP was very well controlled. The possible reasons for the iris incarceration are related to the following factors: (1) If the preoperative IOP is excessively high (>30 mmHg), the intraoperative and postoperative aqueous humor is flushed out very fast, resulting in iris surging and affixing to the thinner TDM area and PAS. Therefore, the preoperative IOP for CLASS should be controlled at ≤30 mmHg as much as possible, so that the intraoperative aqueous humor can have a stable outflow until the IOP is lowered to about 10 mmHg before suturing the scleral flap. (2) After the surgery, massaging the eyeballs or external stimuli such as severe cough can cause rupture of internal Schlemm's canal wall and trabecular meshwork besides iris incarceration. Thus, patients are advised not to compress or strain eyes after the surgery. During the follow-up, no severe complications were observed in the CLASS group. In the Trab group, a thin-wall bleb was found in five eyes, with no discomfort or increased IOP. Meanwhile, four eyes with uncontrolled IOP underwent trabeculectomy with cataract surgery owing to declined VA caused by lens opacity, and another two eyes with uncontrolled IOP were treated with trabeculectomy. Fortunately, the IOP was well controlled in all six eyes. In addition, one eye in the Trab group suffered low-tension maculopathy, which resulted from postoperative excessive filtrating blebs caused by intraoperatively prolonged application of the mitomycin sponge. The low-tension maculopathy gradually recovered after injecting autologous blood into the filtration bleb.

This study had some limitations such as the single-center design, single ethnic population, small cohort of patients, and short-term follow-up. In the future, a more rigorous, large-scale, multicenter study encompassing patients of different ethnicities and with a longer follow-up should be performed to further verify and validate the effect of CLASS.

## 5. Conclusion

As a modification of nonpenetrating deep sclerectomy, CLASS was believed to be remarkably less effective compared with trabeculectomy, although CLASS has a better safety profile. In the study, we confirmed that CLASS is an effective and safe treatment modality for POAG, with fewer filtering bleb-related complications and quicker visual recovery in the early postoperative stage than trabeculectomy. The efficacy of lowering intraocular pressure was similar for both procedures.

## Figures and Tables

**Table 1:** Patients' information of the two groups before the surgery.

Group	*n*	Gender		Age (years)	IOP (mmHg)	BCVA (LogMAR)	Number of medications
		M	F				
CLASS	30	18	6	46.62 ± 12.43	36.43 ± 6.62	0.24 ± 0.27	3.57 ± 1.19

Trab	47	25	13	46.34 ± 14.36	37.87 ± 5.74	0.27 ± 0.31	3.60 ± 0.99

*χ* ^2^/*t*	—	0.59		0.56	1.01	0.43	0.12

*P*	—	0.44^b^		0.58^a^	0.32^a^	0.67^a^	0.91^a^

M: male, F: female, IOP: intraocular pressure, BCVA: best-corrected visual acuity, and LogMAR: logarithm of minimal angle of resolution. ^a^*P* values were calculated using the unpaired *t*-test; ^b^*P* value calculated using the chi-squared test.

**Table 2:** Changes in BCVA in two groups before and after the surgery.

Group	Time			Time effect		Group effect		Interaction effect	
	Before	After 1 week	After 1 month	*F*	*P*	*F*	*P*	*F*	*P*
CLASS (*n* = 30)	0.24 ± 0.27	0.28 ± 0.22	0.27 ± 0.24						
Trab (*n* = 47)	0.27 ± 0.32	0.44 ± 0.33	0.31 ± 0.31						
*P*	0.67	0.02	0.55	23.14	<0.001	1.39	0.24	12.33	<0.001

BCVA: best corrected visual acuity, LogMAR: logarithmic minimum angle of resolution. Repeated measure ANOVA was used and the Mauchly's sphericity test P<0.01, so the Greenhouse–Geisser estimation was conducted to calibrate the results. The interaction between group effect and time effect was statistically significant, so a simple effect analysis of the data was further performed

**Table 3:** Changes in IOP (mmHg) and number of medications (*n*) in two groups before and after the surgery.

	Time							Time effect		Group effect		Time *∗* Group	
	Preoperation	After 1 wk	After 1 mo	After 3 mo	After 6 mo	After 12 mo	After 24 mo	*F*	*P*	*F*	*P*	*F*	*P*
IOP													
CLASS	36.43 ± 6.62	11.94 ± 3.09	13.97 ± 3.33	14.83 ± 3.68	15.10 ± 3.38	15.24 ± 3.37	15.38 ± 3.74						
Trab	37.87 ± 5.74	12.87 ± 3.11	15.21 ± 2.75	15.74 ± 2.47	15.83 ± 2.69	15.93 ± 3.36	16.34 ± 3.25	360.11	<0.001	0.28	0.42	0.08	0.99

Medications													
CLASS	3.57 ± 1.19	—	—	0.83 ± 1.17	1.03 ± 1.32	1.24 ± 1.50	1.31 ± 1.54						
Trab	3.60 ± 0.99	—	—	0.85 ± 0.98	1.11 ± 1.23	1.28 ± 1.26	1.30 ± 1.32	108.11	<0.001	0.047	0.830	0.048	0.981

IOP: intraocular pressure. Repeated-measures ANOVA was used and the Mauchly sphericity test *P* < 0.1, so the Greenhouse–Geisser estimation was conducted to calibrate the results.

**Table 4:** Success rate over time between the two groups *n* (%).

Time	Parameters	*n*	CLASS	*n*	Trab	*χ* ^2^	*P*
			Success rate		Success rate		
After 6 months	Complete	29	24 (82.8)	46	38 (82.6)	<0.001	0.99
	Qualified	29	29 (100)	46	44 95.7)	1.30	0.26
After 12 months	Complete	29	17 (58.6)	45	27 (60.0)	0.14	0.91
	Qualified	29	27 (93.1)	45	42 (93.3)	<0.001	0.97
After 24 months	Complete	29	15 (51.7)	44	21 (47.7)	0.11	0.74
	Qualified	29	25 (86.2)	44	37 (84.1)	0.06	0.81

*P* values were calculated using the chi-squared test.

**Table 5:** Postoperative complications in CLASS versus Trab groups.

Complication	*n* (%)		*P*
	CLASS group (*n* = 30)	Trab group (*n* = 47)	
Shallow anterior chamber	0 (0)	10 (21.3)	<0.001
Hyphema	0 (0)	4 (8.5)	0.15
Ciliary body detachment	1 (3.3)	0 (0)	0.39
Choroid detachment	0 (0)	5 (10.6)	0.14
Low-tension maculopathy	0 (0)	1 (2.1)	1.00
Peripheral anterior synechiae	9 (30)	0 (0)	<0.001
Iris incarceration	2 (6.7)	0 (0)	0.15
Thin-wall filtrating bleb	0 (0)	5 (10.6)	0.15

*P* values were calculated using Fisher's exact test.

## Data Availability

The research data used to support the findings of this study are available from the corresponding author upon request.
